# The relationship between Chinese preschool principal leadership styles and teacher leadership: Exploring the mediating effect of psychological capital

**DOI:** 10.3389/fpsyg.2022.1006184

**Published:** 2022-09-30

**Authors:** Limin Zhang, Tingting Wu, Lijia Liu, Ping Ren, Chaopai Lin

**Affiliations:** ^1^Department of Early Childhood Education, School of Education, Guangzhou University, Guangzhou, China; ^2^Huadong Town Central Preschool, Guangzhou, China; ^3^Department of Pedagogy, School of Education, Guangzhou University, Guangzhou, China; ^4^Department of Early Childhood Education, School of Education, Central China Normal University, Wuhan, China

**Keywords:** early childhood education, principal leadership styles, teacher leadership, psychological capital, teacher education

## Abstract

Enhancing teacher leadership is not only one of the approaches to improving teaching and learning, but it is also essential to the success of education reform. Based on leader-member exchange theory, 294 preschool teachers in China were surveyed, and a structural equation model was established to explore the relationship between the participating teachers' principal leadership style, teacher leadership and psychological capital. The findings revealed a significant positive correlation between transformational and transactional leadership styles and preschool teacher leadership. The laissez-faire leadership style had no correlation with preschool teacher leadership. The transformational leadership style and transactional leadership style were significantly and positively correlated with psychological capital, while the laissez-faire leadership style was significantly and negatively correlated with psychological capital. The transformational leadership style can positively influence preschool teacher leadership directly and indirectly through psychological capital; and the transactional leadership style can only positively influence preschool teacher leadership indirectly through the mediating role of psychological capital. Preschool teachers' leadership can neither be directly influenced by a laissez-faire leadership style nor be indirectly influenced through the mediating role of psychological capital.

## Introduction

Since the 1980's, with the emergence of educational reform worldwide, many countries have started to recognize the cultivation and enhancement of teacher leadership as a way to achieve successful educational reform. Teacher leadership has become a key factor in driving change in schools. Scholars have come to realize that the model of relying solely on the school's top leader, the preschool principal, to promote school development is not working as well as expected (Harris and Muijs, 2005). The traditional concept of one-person leadership by the preschool principal has been replaced by a new concept of leadership that recognizes the potential of shared leadership by teachers within the school (Marks and Printy, 2003; Katzenmeyer and Moller, 2009). Moreover, in preschool, the individual leadership of the preschool principal is not sufficient to meet the various developmental needs of the school and the children. Every teacher or member of the organization in the preschool has the authority and responsibility to demonstrate their leadership (Bellibaş et al., 2020). Educational reform can be acted upon successfully only when teachers act (Beachum and Dentith, 2004).

Teacher leadership practices, in other words, involve teachers in decision-making within the school. The scope of this decision-making is not limited to a particular classroom, but covers nine school-wide areas: instructional co-ordination, curriculum development, staff development, evaluation, general school improvement, personnel, rules and discipline, general administration and policymaking (Duke et al., 1980). In these areas, the role of teachers in the decision-making of early childhood curriculum is very important, as curriculum is the core of early childhood education and successful leadership (Yang, 2019). Similarly, teachers are also an important factor in the development of early childhood curriculum (Yu, 2007). The *Basic Education Curriculum Reform Outline (Trial)* issued by China's Ministry of Education in 2001 states that preschool teachers should make practical work plans and implement them flexibly according to the practical situation of the children in the class. During the transformation phase of the curriculum, each local preschool has its own characteristics, and each teacher and child in each school is different. Preschool teachers, as implementers of the early childhood curriculum, are in a better position to develop and implement appropriate curricula because they are more aware of the physical and psychological characteristics of their own children and their curriculum practices (Wei and Cheng, 2022). At the same time, this requires teachers to shift from their previous role as curriculum implementers, to give full play to their leadership in curriculum development, to flexibly adjust and optimize the curriculum based on children's authentic feedback, and to build a truly child-centered curriculum.

### Teacher leadership

Harris and Muijs (2005) defined teacher leadership as the ability of a community of teacher learners to contribute and influence others to improve educational practices. York-Barr and Duke (2004) defined teacher leadership as the process through which teachers influence members of the school community to improve their teaching and learning. Enhancing teacher leadership not only supports the embedding of educational reform but also promotes the process of teachers' professional learning and development (Taylor et al., 2011). Through collaborative leadership, early childhood education professionals can lead the reform of pedagogy that shapes and improves their professional practice (Hallet, 2013). In an atmosphere where “teacher leadership” is emphasized and promoted, there will be greater collaboration between teachers, a greater desire for teachers to have a voice in the improvement of teaching and learning, and a more proactive focus on improving and changing teaching practice. In the professional learning community, teacher professional development is supported through peer observation, team teaching and reflective dialogue (Chow, 2016). As long as teachers have the appropriate support, they can lead innovation, build professional knowledge, and develop their leadership capacity and influence and practice in their schools (Frost, 2012). The cultivation and improvement of teacher leadership can change the traditional role positioning and understanding of teachers in a timely manner and make teachers realize that they can also be nonpower “leaders” with a positive influence on others.

### Principal leadership style

It is obvious that the improvement of teacher leadership requires the unremitting efforts of individual teachers, but it will also be affected by other factors. Peer relationships are a key factor affecting teacher leadership (Margolis, 2012; Fairman and Mackenzie, 2015). High trust and positive working relationships among peers and with administrators can increase teachers' willingness to support other teachers in cooperation (Silva et al., 2000). In addition, preschool principals have played an important role in influencing the development of teacher leadership, including whether preschool principals have a clear understanding of the role of leaders, whether they have accepted the existence of teacher leaders and recognized the value of their presence rather than a threat, and whether they have encouraged good teachers to become leaders (Buckner and McDowelle, 2000). The overly authoritarian or permissive leadership styles of preschool principals are not conducive to the development of teacher leadership (Thornton, 2010). Highly supportive preschool principals express their expectations of improving teaching to teacher leaders in repeated communication and regard teacher leaders as useful teaching resources. They also support the development of teacher leaders by adopting strategies such as “expecting teachers to communicate with teacher leaders” in communication (Mangin, 2007).

Influenced by a hierarchical management structure, the management system of preschool in China is led by the preschool principal, which means the preschool principal is responsible for dealing with the mission of the school, the training plan and the appointment of teachers and other major issues and decisions (Jiang et al., 2016). The preschool principals actually have full leadership over the preschool. In the process of managing the preschool and creating organizational culture, the preschool principals' own leadership behavior will affect the psychological state and working state of teachers. For example, preschool principals influence teachers' job satisfaction and leadership through their authority degree of openness (Wang and Xia, 2020). The process by which the principal delegates and discloses his or her authority to the preschool teachers is essentially the principal's adoption of an open and inclusive leadership style to enhance the leadership of the preschool teachers.

According to *full-range leadership theory* (FRLT), the latest paradigm in leadership style theory, there are three types of leadership styles: transformational, transactional, and laissez-faire. These three leadership styles basically cover the main aspects of modern leadership styles (Avolio and Bass, 2001).

Burns (1978), based on Maslow's hierarchical needing theory, defined the transformational leadership style as a leader who encourages the members of an organization to pursue higher-level work goals through his or her noble moral accomplishments and outstanding leadership. Bass (1995) defined it as the leader giving individual care and intellectual stimulation to the members of the organization through his or her unique charisma and personal characteristics to improve the work involvement of the members of the organization and the work performance of the whole team. By sharing positive visions, transformational leaders internalize the values of their subordinates and urge them to pursue higher goals and objectives beyond immediate interests (Howell and Avolio, 1993). In the context of education, Leithwood (1994) clearly proposed that the transformational leadership style in schools could promote teachers' identification with organizational goals by building a cooperative institutional culture, motivating teachers to develop continuously, and ultimately achieving the development and reform of schools. Transformational leadership, according to Peng et al. (2022), enhanced teachers' work satisfaction through PLCs. Additionally, some studies have indicated that the transformational leadership style of preschool principals can improve teachers' collective efficacy (Dussault et al., 2008), stimulate teachers' professional learning and motivation (Thoonen et al., 2011), and promote the development of teacher leadership (Li and Liu, 2020). Under the transformational leadership style of the preschool principal, teachers are motivated and prepared to assume the responsibility of professional development and the management of instructional leadership (Printy et al., 2009).

The transactional leadership style was first proposed by Burns (1978). According to his perspective, a transactional leadership style refers to leaders selectively providing subordinates with appropriate support and remuneration after understanding their working abilities to meet their material needs for survival and help them successfully complete their tasks. In an equal exchange, subordinates need to be paid for their labor to receive the corresponding reward and support promised by their leaders. Therefore, the transactional leadership style places more emphasis on the exchange of interests or resources between leaders and subordinates. Later, Bass (1995) further generalized the view of this leadership style as focusing on the exchange of resources and the rules of reward and punishment between the leader and the subordinate. Transactional leadership style, which, in essence, is a transactional process, urges subordinates to work hard with the help of spiritual incentives and material rewards. The transactional leadership style emphasizes the exchange of benefits and material incentives, but it is crucial to establish a reward and punishment incentive mechanism and to provide an indispensable material guarantee for improving teachers' innovative teaching behavior. When teachers are under the management of a leader with a transactional leadership style, they know that future rewards or punishments depend on whether their behaviors meet expectations. Therefore, they need to achieve goals through self-leadership and intrateam leadership (Marshall et al., 2012).

The laissez-faire leadership style was first defined by Lewin to describe a leader who rarely uses his or her managerial authority when leading a team and habitually takes a nondirective and dismissive attitude toward subordinates, rarely giving direction to team members about the team's tasks and goals. Bass later put forward that the laissez-faire leadership style is essentially a kind of leadership behavior in which the leader allows subordinates to freely develop without assuming relevant management actions and in which subordinates are often in a state of being overwhelmed. Some categorize the laissez-faire leadership style as the antithesis of the transformational leadership style and transactional leadership style in which the leader-subordinate deal is made (Bass and Avolio, 1993). However, the laissez-faire leadership style is extremely common in the actual workplace (Aasland et al., 2009). Some scholars argue that because laissez-faire leaders are not involved in the work of their subordinates, they are motivated to adopt assertive behaviors such as making demands, expressing emotions, and displaying assertiveness to fill the lack of leadership influence and maximize subordinates' own leadership (Deluga, 1990). However, the negative results of the laissez-faire leadership style are still predominant. The laissez-faire leadership style of preschool principals negatively affects teachers' collective efficacy (Dussault et al., 2008), job performance and satisfaction (Imhangbe et al., 2019). Moreover, the laissez-faire leadership style affects the quality of interpersonal relationships within the school and is detrimental to conflict resolution among teachers (Chandolia and Anastasiou, 2020). As laissez-faire leaders relinquish their responsibilities, subordinates may compete for power and influence abdicated by the laissez-faire leader, which can lead to interpersonal tensions (Deluga, 1990). Teacher leadership cannot be developed and grow in a laissez-faire environment.

Therefore, the following hypotheses were proposed:

Hypothesis 1a: A transformational leadership style has a significant positive impact on preschool teacher leadership.
Hypothesis 1b: A transactional leadership style has a significant positive impact on preschool teacher leadership.
Hypothesis 1c: A laissez-faire leadership style has a significant negative impact on preschool teacher leadership.

### Psychological capital

In the process of teaching in preschool, teachers confront a variety of difficult jobs and assignments, as well as a variety of obstacles. They are easily defeated by setbacks if their psychological quality is weak. Therefore, psychological quality is one of the key factors for preschool teachers to be competent in their jobs. Luthans argues that psychological capital is a comprehensive source of energy that combines multiple positive psychological states, including self-efficacy, hope, optimism and resilience, which can enhance the quality of one's work and life (Luthans et al., 2005) and widely influence individuals' attitudes and behaviors (Avey et al., 2010; Peng et al., 2013). Different leadership styles have a great impact on the psychological capital of employees and the whole team. There is a significant positive correlation between sincere leadership and employee psychological capital (Woolley et al., 2011). Within the school context, sincere leadership by preschool principals significantly fosters positive psychological capital in teachers (Feng-I. F, 2016). A transformational leadership style positively predicts employees' psychological capital, while psychological capital mediates the impact of transformational leadership on engagement (Yongzhan and Li, 2018) and is also positively associated with team performance (Rebelo et al., 2018). By constructing a vision, transformational leaders are able to help employees clarify their goals and directions and recognize the value and meaning of the work they are doing; these goals and visions, in turn, inspire enthusiasm and increase hope and confidence in the future (Helland and Winston, 2005). Under a laissez-faire leadership style, employees do not receive effective support and motivation from their leaders' behaviors and do not cultivate a sense of trust and confidence in the organization (Baig et al., 2021), thus resulting in a negative organizational environment. Such a leadership style will have a negative impact on the mental health of organization members (Toor and Ofori, 2010).

Therefore, the following hypotheses were proposed:

Hypothesis 2a: A transformational leadership style has a significant positive impact on the psychological capital of preschool teachers.
Hypothesis 2b: A transactional leadership style has a significant positive impact on the psychological capital of preschool teachers.
Hypothesis 2c: A laissez-faire leadership style has a significant negative impact on the psychological capital of preschool teachers.
Hypothesis 3: The psychological capital of preschool teachers has a significant positive impact on preschool teacher leadership.

### Theoretical framework

Leader-member exchange theory (LMX) provides a good theoretical perspective for studying and analyzing the relationship between preschool principal leadership styles, teacher leadership and psychological capital. The uniqueness of this theory lies in its research focus on the dynamic exchange relationship between the leader and the members of the organization and on the mechanisms by which this dynamic exchange relationship affects the work attitudes and behaviors of the members of the organization. LMX is based on social exchange theory, in which the interaction of interpersonal relationships is a central theme. LMX refers to the quality of the exchange relationship between leaders and followers based on trust, respect and obligation. In exchange for a comprehensive view of the leader's support and motivation, the employee will reward the leader with respect, trust, and adequate feedback, thus establishing a relationship with a high level of mutual esteem (Graen and Uhl-Bien, 1995). Individuals who develop high-quality relationships with their leaders will be attached psychologically to their work group (Pan and Lin, 2016). A high-quality leader-member exchange relationship brings good exchange results for leaders and organizational members. Because of the positive effects of a high-quality leader-member exchange relationship, it is beneficial to maintain a positive exchange relationship between leaders and organizational members for a long time (Wilson et al., 2010). From the perspective of leadership style, the transformational leadership style directly affects the quality of the leader-member exchange relationship (Wang et al., 2005; Vermeulen et al., 2022). This study suggests that there is a unique leader-member exchange relationship between the leadership styles of preschool principals, teacher leadership and the psychological capital of preschool teachers. Different leadership styles of preschool principals have different influences on the psychological state and behavioral practices of preschool teachers. When preschool teachers receive resources and support from preschool principals with different leadership styles, they enter into an exchange relationship with the preschool principals. Thus, the various leadership practices that preschool teachers exhibit at work involve feedback and exchange with the resources provided by preschool principals with different leadership styles.

Therefore, the following hypotheses were proposed:

Hypothesis 4a: A transformational leadership style affects teacher leadership through the mediating role of preschool teachers' psychological capital.
Hypothesis 4b: A transactional leadership style affects teacher leadership through the mediating role of preschool teachers' psychological capital.
Hypothesis 4c: A laissez-faire leadership style affects teacher leadership through the mediating role of preschool teachers' psychological capital.

### Present research

Different leadership styles influence subordinates' work attitudes and behavioral practices (Avolio et al., 1999). The current management system of preschools in China is the principal responsibility system, in which the principal is responsible for all people, things, and objects in the preschool. The different leadership styles adopted by the preschool principal have an impact on the psychological state of preschool teachers and a range of behavioral practices inside and outside the classroom. At present, early childhood education in China is receiving more and more attention from the government and society, and building a high-quality teacher team and improving the quality of early childhood education are the most important tasks of the current education reform. The training and enhancement of teacher leadership is one of the main ways of professional development of teachers, which is not only conducive to the continuous learning of individual teachers, but also can strengthen the professional construction of the teaching team. But most of the research on teacher leadership in the Chinese context has been conducted on primary and secondary school teachers and university teachers, but less on preschool teacher leadership. The research has focused on the current situation of teacher leadership and its influencing factors, but there is a lack of research to explore the deeper mechanisms underlying preschool teacher leadership, especially the relationship between the leadership style of preschool principals and preschool teacher leadership.

Therefore, this study aims to:

1. Investigate the current situations of preschool teacher leadership, psychological capital, and preschool teachers' perceived leadership styles of principals and the differences between the three with respect to different demographic variables.
2. Explore the correlation between principal leadership style, teacher psychological capital and teacher leadership and analyse the influence of different types of preschool principal leadership styles on preschool teacher leadership based on theories and data.
3. Establish a structural equation model to test the mediating role of psychological capital in different preschool principal leadership styles and preschool teacher leadership.

**Figure 1:**
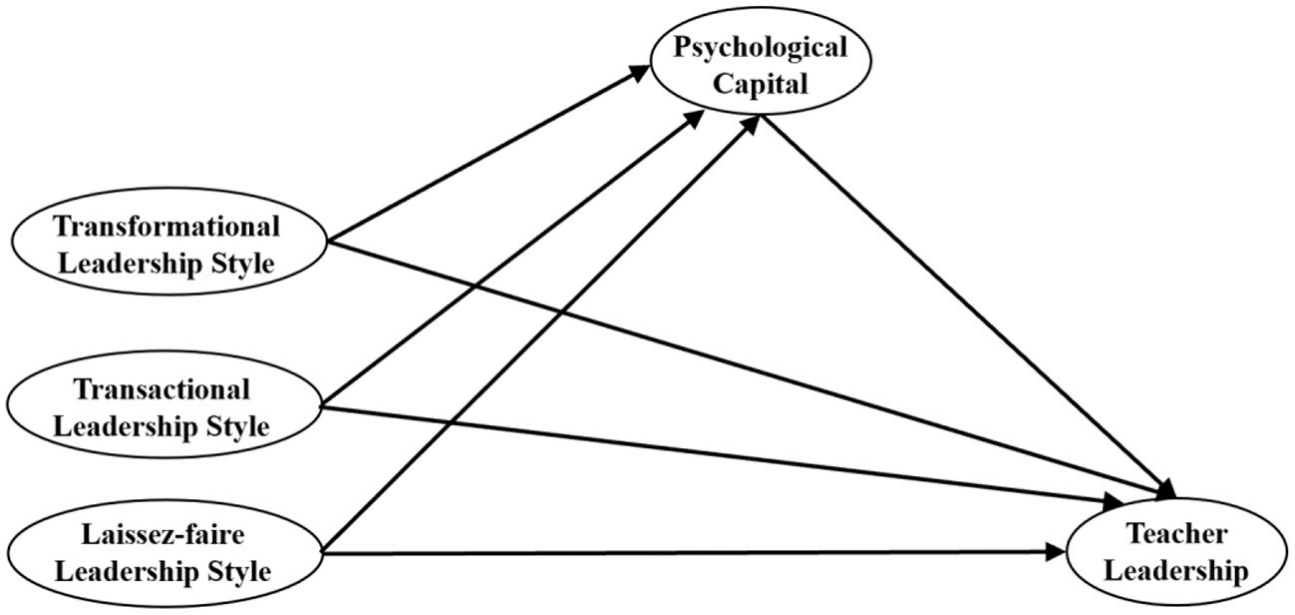
Hypothetical model.

The hypothetical model of the present study is shown in Figure 1.

## Materials and methods

### Participants

In this study, preschool teachers from Guangdong Province in China was selected as the research participants. Three hundred twenty-seven pieces of data were collected, and 294 valid questionnaires were recovered, with a recovery rate of 89.91%. Specific demographic information of the sample, including teachers' age, gender, position, educational background, seniority and types of preschools, is shown in Table 1.

**Table 1:** Demographics of participants (*N* = 294).

**Demographic characteristic**		***N***	**%**
Gender	Male	10	3.4
	Female	284	96.6
Age	<25	172	58.5
	26–30	70	23.8
	31–35	18	6.1
	36–40	16	5.4
	40–45	14	4.8
	>45	4	1.4
Educational background	High school or less	5	1.7
	Junior college	87	29.6
	Bachelor degree	189	64.3
	Master's degree or above	13	4.4
Seniority	Under 3 years	194	66
	4–5 years	49	16.7
	6–10 years	23	7.8
	11–15 years	11	3.7
	16–20 years	8	2.7
	Above 20 years	9	3.1
Type of preschool	Public	197	67
	Private inclusiveness	55	18.7
	Private	24	8.2
	Others	18	6.1
Position	Assistant teacher	161	54.8
	Head teacher	85	28.9
	Grade/teaching and research group leader	10	3.4
	Administrative positions	12	4.1
	Others	26	8.8

### Measures

#### Teacher leadership scale

This study uses the Teacher Leadership Scale (TLS) developed by Chinese scholars Wang and Xia (2020) to measure the leadership of preschool teachers. The scale has 19 items in total, including four dimensions, namely leading teaching and professional development; characteristics of teacher leaders; participating in school-wide decision-making; diversity and continuous improvement. The scale is based on a Likert-6 scale, ranging from 1 for “strongly disagree” to 6 for “strongly agree.” The overall Cronbach's α of the scale was 0.926, and the internal consistency coefficients of the four dimensions were 0.91, 0.92, 0.87 and 0.87 respectively, indicating that the scale had high reliability.

#### Principal leadership styles

The Multifactor Leadership Questionnaire (MLQ-5X) compiled by Avolio and Bass (2001) and the Paternalistic Leadership Scale (PLS) compiled by Cheng et al. (2014) were used to measure the types of leadership styles of preschool principals. The MLQ-5X scale consists of three subscales with 36 items, Transformational Leadership style (20 items), Transactional Leadership style (8 items), and Laissez-Faire Leadership style (8 items). The Likert-5 grading method was adopted for the scale, ranging from “strongly disagree” to “strongly agree.” The overall Cronbach's α of the scale was 0.937, and the Cronbach's α values of the four subscales were 0.975, 0.83, and 0.95 respectively, indicating that the scale has high reliability.

#### Psychological capital questionnaire

The Psychological Capital Questionnaire (PCQ-24) prepared by Luthans et al. (2007) was adopted in this study. Some words of the Questionnaire were modified appropriately, for example, “company” was changed to “preschool,” etc. Therefore, it is suitable to be tested in preschool teachers. A Likert scale of 6 points was adopted, from 1 “strongly disagree” to 6 “strongly agree.” A higher score indicates a higher level of psychological capital. The Cronbach's α of the overall scale was 0.90, showing that the scale has good reliability.

### Statistical analysis

First of all, the single-factor ANOVA test was used to test the differences in preschool teacher leadership and teacher psychological capital in teachers' seniority and position. Secondly, the correlation and relationship among these three variables of preschool teacher leadership, preschool principal leadership styles and teacher psychological capital were tested. After the significant correlation and regression of the three variables were verified, finally, a structural equation model with Maximum Likelihood Estimation was used to verify the mediating role of psychological capital between different preschool principal leadership styles and preschool teacher leadership.

SPSS(Version 25.0) and Mplus (Version 8.0) were used for data analysis.

## Results

### Description statistics and correlation matrix

The mean (*M*), standard deviation (*SD*) of each variable and the correlation coefficient between variables are shown in Table 2. The results show that the scores of transformational leadership style (*M* = 3.97, *SD* = 0.75) and transactional leadership style (*M* = 3.75, *SD* = 0.65) are both higher than the theoretical median value of 3, indicating that the two kinds of leadership styles perceived by preschool teachers are at a high level. But the scores of laissez-faire leadership style (*M* = 2.44, *SD* = 1.13) is lower than the theoretical median value of 3, indicating that the two kinds of leadership styles perceived by preschool teachers are at a low level. The score of teacher leadership (*M* = 4.92, *SD* = 0.62) is higher than the theoretical median value of 3, indicating that the preschool teacher leadership is at a high level. The score of psychological capital (*M* = 4.35, *SD* = 0.61) is higher than the theoretical median value of 3, indicating that the psychological capital of preschool teachers is at a high level.

**Table 2:** The correlational coefficients of all variables (*N* = 294).

		**M**	**SD**	**1**	**2**	**3**	**4**	**5**
1	Transformational Leadership Style	3.97	0.75	1				
2	Transactional Leadership Style	3.75	0.65	0.686^**^	1			
3	Laissez-Faire Leadership Style	2.44	1.13	−0.311^**^	−0.070	1		
4	Teacher Leadership	4.92	0.62	0.600^**^	0.534^**^	−0.088	1	
5	Psychological Capital	4.35	0.61	0.565^**^	0.422^**^	−0.389^**^	0.552^**^	1

* *p* < 0.05,

** *p* < 0.01,

*** *p* < 0.001.

Preschool teacher leadership was positively correlated with transformational leadership style (*r* = 0.600, *p* < 0.001) and transactional leadership style (*r* = 0.534, *p* < 0.001). There is no correlation between laissez-faire leadership style and preschool teacher leadership (*r* = 0.088, *p* > 0.05). Psychological capital of preschool teachers was positively correlated with transformational leadership style (*r* = 0.565, *p* < 0.01) and transactional leadership style (*r* = 0.422, *p* < 0.01), while the psychological capital of preschool teachers was negatively correlated with laissez-faire leadership style (*r* = −0.389, *p* < 0.01). There was a significant positive correlation between preschool teacher leadership and psychological capital (*r* = 0.552, *p* < 0.01).

### Variance analysis

The mean difference test was conducted for the two variables (teacher leadership, psychological capital). The results are shown in Table 3. In terms of teacher leadership, there were significant differences for seniority, *F* = 4.876, *p* < 0.01. *Post-hoc* analysis using the Scheffé *post-hoc* criterion for significance indicated that the leadership of preschool teachers with 3 years or less of seniority (*M* = 4.80, *SD* = 0.62) was significantly lower than who with more than 4 years of seniority (*M* = 5.14, *SD* = 0.59), and reaches the peak within the range of 11–15 years of seniority (*M* = 5.33, *SD* = 0.46). There were significant differences for position, *F* = 3.216, *p* < 0.01. *Post-hoc* analysis showed that the score of assistant teachers (*M* = 4.82, *SD* = 0.60) was significantly lower than that of teachers in more senior positions. The leadership level of administrative teachers was the highest among all the teachers (*M* = 5.29, *SD* = 0.41).

**Table 3:** Variance analysis (*N* = 294).

	**Variable**	**Group**	***M***	***SD***	***F***
Teacher leadership	Seniority	Under 3 years	4.80	0.62	4.876^***^
		4–5 years	5.14	0.59	
		6–10 years	5.09	0.49	
		11–15 years	5.33	0.46	
		16–20 years	5.19	0.56	
		Above 20 years	5.18	0.46	
	Position	Assistant teacher	4.82	0.60	3.216^***^
		Head teacher	5.01	0.63	
		Grade/teaching and research group leader	5.17	0.48	
		Administrative positions	5.29	0.41	
		Others	5.00	0.67	
Psychological capital	Seniority	Under 3 years	4.23	0.62	5.569^*^
		4–5 years	4.45	0.50	
		6–10 years	4.69	0.54	
		11–15 years	4.89	0.42	
		16–20 years	4.46	0.59	
		Above 20 years	4.57	0.50	
	Position	Assistant teacher	4.26	0.63	3.508^*^
		Head teacher	4.40	0.55	
		Grade/teaching and research group leader	4.80	0.51	
		Administrative positions	4.71	0.54	
		Others	4.36	0.61	

* p < 0.05,

** p < 0.01,

*** p < 0.001.

In terms of psychological capital, there was significant difference for seniority, *F* = 5.569, *p* < 0.05. *Post-hoc* analysis showed that the score of preschool teachers with 11–15 years of seniority (*M* = 4.89, *SD* = 0.42) was highest. There was significant difference for position, *F* = 3.508, *p* < 0.05. *Post-hoc* analysis indicated that score of assistant teachers (*M* = 4.26, *SD* = 0.63) was significantly lower than that of teachers in more senior positions.

### Mediation test of psychological capital between principal leadership styles and teacher leadership

**Figure 2:**
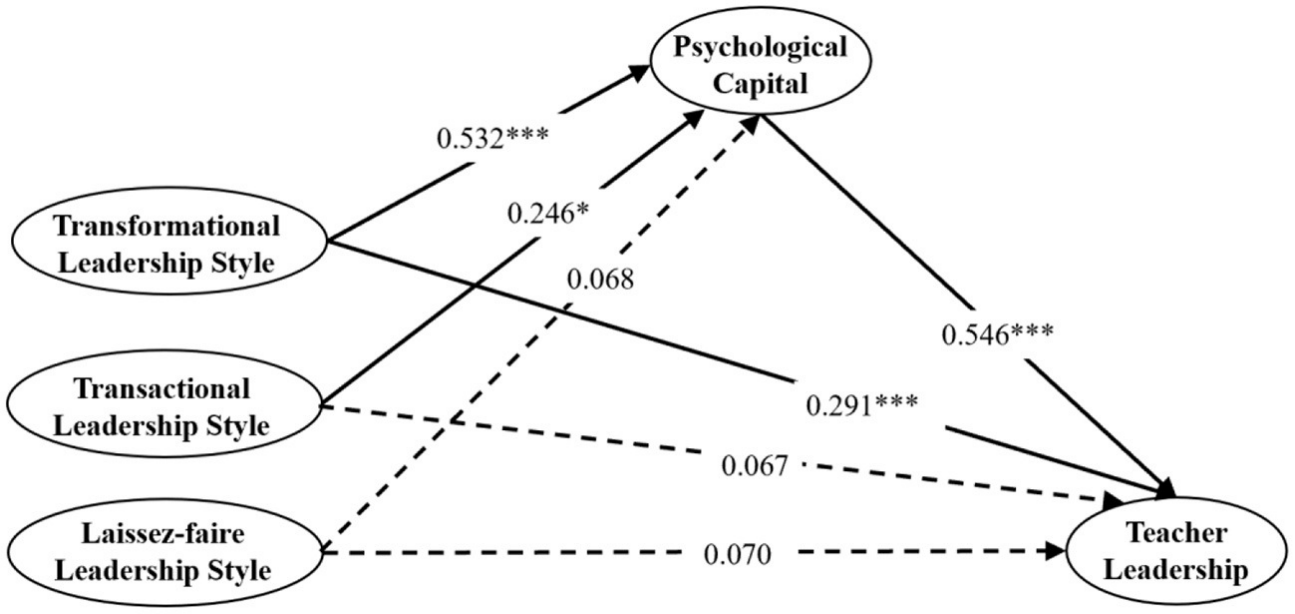
Psychological capital as a mediator in the associations between principal leadership styles and teacher leadership.*p < 0.05, **p < 0.01, ***p < 0.001. Dotted lines indicate non-significant paths.

The purpose of this study is to explore the relationship between the principal leadership style, the preschool teacher leadership and the psychological capital of preschool teachers, which focusing on the mediating effect of psychological capital. On this basis, a structural equation model was constructed, and the model results are shown in Figure 2.

According to (Hu and Bentler, 1999), when the model fit index is χ ^2^/df ≤ 3, CFI ≥ 0.90, TLI ≥ 0.90, RMSEA ≤ 0.08 and SRMR ≤ 0.80, the model is considered to be a good fit. The fitting index of the model is *x*^2^ = 6359.37, *df* = 2332, RMSEA = 0.073, CFI = 0.905, TLI = 0.918, SRMR = 0.047. Transformational leadership style has a significant positive impact on psychological capital (*β* = 0.550, *p* < 0.001) and teacher leadership (*β* = 0.316, *p* < 0.001). Transactional leadership style has a significant positive impact on psychology capital (*β* = 0.207, *p* < 0.05). Psychological capital has a significant positive impact on teacher leadership (*β* = 0.532, *p* < 0.001). Transactional leadership style (*β* = 0.061, *p* < 0.05) has no significant impact on teacher leadership. Laissez-faire leadership style has no significant impact on teacher leadership (*β* = −0.013, *p* < 0.05) and psychology capital (*β* = 0.113, *p* < 0.05).

To examine the indirect effects of psychology capital, mediation analysis based on 5,000 bootstrapping samples was conducted. The results were shown in Table 4. In the Hypothesis 4A pathway (Transformational leadership style—Psychology capital—Teacher Leadership), the mediating effect quantity was 0.204, *p* < 0.001, the 95% confidence interval was [0.107, 0.352], the mediating effect ratio (ab/c) was 48.2% (0.204/0.423). Therefore, it can be assumed that psychological capital plays a significant intermediary role in transformational leadership style and teacher leadership. In the Hypothesis 4B pathway (Transactional leadership style—Psychology capital—Teacher leadership), the mediating effect quantity was 0.253, *p* < 0.001, the 95% confidence interval was [0.228, 0.449], the association between transactional leadership style and teacher leadership is non-significant. Therefore, psychological capital fully mediated the relationship between transactional leadership style and teacher leadership. In the Hypothesis 4C pathway (Laissez-Faire leadership style—Psychology capital—Teacher Leadership), all paths are not significant and there is no mediating effect.

**Table 4:** Mediation analysis of psychological capital on the associations between principal leadership styles and teacher leadership.

			**Mediation analysis**				
						**95% CI**	
**Hypothesis**	**Independent variable**	**Dependent variable**	**Mediation variable**	**Estimates (SE)**	***p***	**Lower**	**Upper**
4A	Transformational leadership style	Teacher leadership	Psychological capital	0.204(0.061)	0.000	[0.107, 0.352]	
4B	Transactional leadership style			0.253(0.037)	0.000	[0.228, 0.449]	
4C	Laissez-faire leadership style			−0.003(0.021)	0.891	[−0.045,0.037]	

## Discussion

### Teacher leadership

On the whole, preschool teacher leadership is at an above-average level. First, since comprehensive education reform was launched in most areas of China, teacher-related policies in early childhood education reform have been considered a decisive factor in improving the quality of early childhood education, among which improving preschool teacher leadership is one of the important factors for the success of education reform (Wang and Ho, 2020). In this context, an increasing number of preschool teachers have gradually realized that teacher leadership has a positive influence and that all teachers could be the subject of exercising leadership. Second, preschool teacher leadership is also related to the management style of the preschool principal. The authority openness of the preschool principal is one of the key factors affecting the leadership of preschool teachers (Wang, 2018). When the principal recognizes the importance of teacher leadership to the development of the preschool, he or she will give much support and guidance to the performance of teacher leadership, delegate more decision-making and management rights to teachers, and encourage teachers to exert their positive influence (Devos et al., 2014). LMX theory emphasizes the dynamic relationship between managers and organization members (Graen and Uhl-Bien, 1995). Transferred to the organizational relationships in a preschool, when the principal delegates more leadership to teachers, it is beneficial for teachers to develop good role perceptions, take on more responsibility for additional roles, and use their expertise and competence to exert positive, nonpowerful influence over children, other teachers, and the whole school.

Preschool teacher leadership increases with years of teaching seniority and is lowest for teachers with <3 years of teaching experience. With the increase in teaching seniority, teacher leadership also gradually improves, reaching the highest level at 11–15 years of teaching seniority. Although it declines later, the range is not significant. According to the five-stage theory of teacher professional development proposed by Berliner (1988), teachers with <3 years of teaching experience are at the stage of novice and advanced beginners, who are trying to adapt themselves to the new environment and pay less attention to teaching and the decision-making of school affairs. With the increase in teaching seniority, novice teachers become experienced teachers, and their focus gradually shifts to activities and children at schools. They become more competent and have more time to pay attention to the decision-making of school affairs. With the accumulation and precipitation of teaching experience, the teachers with more seniority receive more attention from the principal and have more say in the decision-making of school affairs, which can effectively develop their leadership.

There is a significant difference in the leadership of preschool teachers in terms of their positions. It is shown that assistant teachers have the lowest scores, while the teachers of administrative positions, such as deputy principals and grade leaders, have the highest scores. Rönnerman et al. (2017) described teachers such as deputy principals and grade leaders as positionally between the principal and the staff and philosophically as a leader among peers as a “middle leader.” Compared with preschool principals, middle leaders in preschools bear more responsibility to manage the whole preschool, such as the grades or the teaching and research groups. They are both managers and executors. Therefore, the improvement of middle-level teachers' leadership is the result of the accumulation of continuous experience in daily management and execution processes.

### Principal leadership styles

Numerous studies of principal leadership styles have shown that the transformational leadership and transactional leadership styles are most frequently exhibited among the school leadership styles, while the laissez-faire leadership style is less common (Li, 2015; Ballaschk et al., 2017; Chandolia and Anastasiou, 2020; Kirkiç and Balc, 2021). In this research, the transformational leadership style of principals scored the highest on the “intellectual stimulation” dimension. Bass and Avolio (1993) defined “intellectual stimulation” as the leader articulating new ideas that prompt followers to rethink conventional practice and thinking. LMX theory suggests that managers will be more supportive of and inspirational to organizational members when they build high-quality leader-member exchange relationships with them (Graen and Uhl-Bien, 1995). When teachers encounter new problems in teaching, such as parental work or scientific research work, the principal encourages teachers to consider problems from new and different perspectives, constantly exercises their problem-solving skills and encourages them to look at problems as a spark of different ideas colliding. In addition, emotional factors are also included as one of the factors affecting transformational leadership style. LMX theory suggests that a high-quality leader-member exchange relationship includes an emotional exchange between managers and organizational members. Preschool is a female-dominated work environment, and the principal and the teachers are mostly women. Actually, female leaders are normally sensitive and considerate and can find the emotional needs of teachers in their work and give them more specialized caring in a timely manner. Similarly, female teachers are also eager for the emotional support and work motivation given by their superior leaders.

Bass and Avolio (1993) defined the two dimensions of the transactional leadership style as the leader providing rewards contingent on performance (contingent reward) and the leader taking corrective action in anticipation of problems (management-by-exception-active). In this study, the score of the “contingent reward” dimension of the transactional leadership style of principals was higher than that of the “management-by-exception-active” dimension. This shows that in the management process of preschools, instead of focusing on “What are the teachers' mistakes? How should I correct them?”, the principal focuses more on “What are the needs of the teachers? What kind of help or reward can I give to the teachers to motivate them to serve the preschool and achieve teaching goals?” LMX theory shows that managers and organizational members are in dynamic exchange relations that influence each other (Graen and Uhl-Bien, 1995). To establish a high-quality leader-member exchange relationship with a team of teachers who have relatively high levels of education, theoretical knowledge and practical experience in teaching, the principal is more inclined to pay attention to the teachers' needs at work and provide them with assistance or incentives in exchange for the teachers bringing more resources to the institution. In addition, due to teachers' high educational and personal quality, there are few behaviors that do not comply with the rules and regulations of the preschool at work; thus, the principal does not need to pay too much attention to the teachers' wrong behaviors.

In this study, the laissez-faire leadership style scores were the lowest, indicating that preschool teachers are less likely to perceive the principal's hands-off approach as effective management today, and Dussault et al. (2008), Kirkiç and Balc (2021) have shown similar results. Preschool teachers regard their principal as a leader who can take on the responsibility of preschool management and make a difference. LMX theory suggests that managers and organizational members interact in a dynamic exchange relationship. This means that when managers give more autonomy to organizational members, they will have a greater sense of identification with the organization, actively participate in the affairs of the organization, and be willing to take on more leadership roles. However, this autonomy does not refer to the laissez-faire leadership style and does not indicate that the principal would ignore management and guidance. The new management style advocates that the principal should give teachers a degree of decentralization and empowerment (Sebastian et al., 2016), but it does not mean that they can stay out of the loop. The director should lead the teachers to participate in the various affairs of the school together and lead all team members to participate in leadership practices with their professional management knowledge and extensive leadership experience.

### Teacher psychological capital

In this study, the psychological capital of preschool teachers is generally above the average level, indicating that preschool teachers can have a positive psychological state at work, remain optimistic and believe that they can solve challenges and difficulties. This is consistent with the results of Fu (2015), Cheng and Gan (2020), and Hong et al. (2022). First, it is related to the object of education that preschool teachers face. Children aged 3–6 are innocent and lively. Children's lovely smiles, pure love and unconditional trust for teachers can, to a certain extent, relieve or even cure their broken hearts due to excessive work pressure and enhance the psychological capital of preschool teachers (Hong et al., 2022). Second, it can also be related to the increasing emphasis on early childhood education in Guangdong Province and across China. In 2018, the Department of Education of Guangdong Province in *The Third Action Plan for The Development of Preschool Education in Guangdong Province (2017-2020)*, clearly indicated that the government will gradually improve the treatment of equal pay for equal work for teachers in public preschools, integrate all preschool education workers into the social security system, and require preschools to buy endowment insurance for them. The state and government departments at all levels should guarantee the material needs of preschool teachers to meet their basic survival and developmental needs so that they can find their great social importance and see the bright prospects of their careers. Therefore, they would have more confidence and hope in this career and could also maintain an optimistic psychological state at work.

### Relationship between preschool principal leadership styles, teacher leadership, and psychological capital

The transformational leadership style of preschool principals can directly and positively influence the leadership of preschool teachers, which is similar to the results of Leithwood and Jantzi (2006) and Li and Liu (2020). When school principals adopt a transformational leadership style to integrate the social and human capital of the school, they incorporate teachers into the decision-making and management of the school (Li and Liu, 2020). In the process of accomplishing the various developmental goals of the preschool, the principal will regard each teacher as an independent individual, encourage the teachers to give full play to their strengths to complete various tasks and allow them to have their own characteristics to exert their own leadership. The transformational leadership style of principals also indirectly affects the leadership of preschool teachers through the mediating role of psychological capital. Transformational leadership emphasizes sharing and the development of teachers' collective capacity (Gkolia et al., 2018), which is often reflected in the professional learning community (PLC) of preschool teachers (Peng et al., 2022). In a professional learning community, the charismatic influence of the director and the motivation of good prospects can make preschool teachers feel a sense of belonging and identification with the organization and maintain a positive psychological state in their work. Transformational leaders also provide teachers with a relaxing, democratic, innovative and transformative work atmosphere that enables them to boldly innovate teaching and actively cooperate with other colleagues. A good psychological state not only allows preschool teachers to concentrate more on their teaching practice but also to be more willing to take on additional roles, actively participate in making decisions and demonstrate their abilities to make a positive impact on the development and management of the school.

It is worth noting that in this study, the direct effect between the transactional leadership style of principals and preschool teacher leadership is not significant, but the mediating effect through psychological capital is significant. This means that the transactional leadership style of principals indirectly affects the leadership of preschool teachers through the intermediary role of their psychological capital. This also echoes the point of view of Marshall et al. (2012) and Li (2015). As a leadership style based on the exchange process, transactional leaders tend to motivate their employees to complete tasks more effectively by setting specific goals or breaking them down into actionable steps (Yongzhan and Li, 2018). In fact, a positive psychological state can help preschool teachers exert their talents more and have a positive influence on children and colleagues in schools. In the step-by-step process of achieving the goals set by leaders, teachers need to explore various new forms of teaching activities and organization with a more positive mindset to continuously improve their work and teaching skills. In addition, teachers are also able to share their unique teaching ideas, plenty of teaching and parenting experience and skills in handling parents with other teachers. Through mutual cooperation and communication among teachers, they can exert a positive influence on their colleagues and increase their sense of efficacy in collective cooperation and their psychological capital, thus promoting the development of preschool teachers' leadership.

There was no direct effect between laissez-faire leadership style and preschool teacher leadership, nor was there an indirect effect on preschool teacher leadership through the mediation of psychological capital. Analyzing the related reasons, we can draw the following conclusions. First, influenced by the hierarchical management structure in China, the management system of Chinese preschools is under the responsibility of the preschool principal (known as “Yuan Zhang” in Chinese), i.e., who is wholly responsible for handling the work of preschool. He or she is responsible for major issues and decisions such as the missions of preschool, training programs, and teachers' appointments (Jiang et al., 2016). Within this framework of responsibility, the principal must fulfill his or her responsibility to manage the whole school's teachers and teaching or be held accountable by the relevant higher authorities.

## Implication

### For preschool principals

As the manager and head of the preschool, the principal's own actions can influence the perceptions and practices of the organization's members. In addition, it may affect the organizational climate of the school as a whole. When the principal is able to actively engage in leadership practices that set an example for teachers, preschool teachers are influenced by the principal's positive behavioral practices and are able to look to the principal and learn from her or his own leadership practices. Therefore, in management and teaching, the principal should actively exert his or her unique charisma and leadership skills to influence preschool teachers' perceptions of leadership practices, to “practice what you preach” and to “teach by example” as management guidelines, and to strive to set an example for preschool teachers with his or her own leadership practices.

When preschool teachers are leaders who have a positive, non-powerful influence on children, colleagues, and even other people in the preschool, they demonstrate the characteristics of leaders who can influence others and lead them to progress. In addition, each teacher is an individual and must shine differently from others in the group. As the manager of the whole team, the principal should be good at discovering the shining points of each teacher, tapping into his or her leadership talents, encouraging teachers to take on leadership roles other than teaching based on their specific situations and personality characteristics, and bringing their professional strengths and authority to bear on other teachers and lead the whole preschool to a higher level of development.

### For preschool teachers

Preschool teachers need to change their role and understanding in a timely manner. Preschool teachers often habitually think of themselves as just a teacher, a follower of the director and management, and do not yet have a clear understanding of the identity of a teacher leader. Wei and Cheng (2022) suggests that having a certain sense of leadership within is a prerequisite for the development of teacher leadership, and that teachers need to recognize that they can also exert positive influence on others as well and can lead team members to grow together. Therefore, the first prerequisite for developing preschool teachers' leadership is to promptly change preschool teachers' orientation and understanding of their own roles, and preschool teachers must realize that they can be non-powerful “leaders” who have positive influence on children, colleagues, and various personnel in the school. This “leader” has nothing to do with position or power, but rather with the recognition and learning of other teachers, school staff and parents for their excellent teaching and professional knowledge.

Teacher leadership is essentially a job-embedded professional development that enables educational reform and instructional improvement through ongoing, site-based professional development (Poekert, 2012). Preschool teachers need to strengthen their professional skills in order to promote leadership. They can rely on a variety of resources provided by the school to enhance their professional talents, such as active participation in professional learning communities (PLCs). In PLCs, educators work together to enhance student learning through inquiry questions; identify goals for educator learning; engage in collaborative learning through formal and informal professional learning strategies such as lesson study, assessment of student work, and peer coaching; reflect on practice; and hold each other accountable for improved practice and outcomes. PLCs are essential to support teacher leaders in overcoming isolation and other challenges they may encounter when assuming leadership responsibilities.

In addition to the above, preschool teachers need to maintain a positive mental state. It is inevitable that preschool teachers will encounter many teaching problems and challenges in their work, and sometimes it is difficult for their professionalism to be recognized by others, and they do not have the support and understanding of parents, or even the understanding and help of colleagues or principals. This requires teachers to adjust their mindset and work status in a timely manner, always have enough confidence and hope in the early childhood education, be able to put in some effort when facing various challenges and problems, believe that they are capable of accomplishing them, and be persistent.

## Limitations and future research

When interpreting the findings of this study, it is important to note its limitations. Firstly, to examine whether there is an effect of leadership style characteristics on the individual characteristics of subordinates in a given organization, the participants should include both individual leaders and all subordinates. The results would be more convincing if nested data between leaders and subordinates could be collected and the correlation between the two sides of the data could be demonstrated.

Secondly, the sample size in this study was somewhat limited, and the findings could not be generalized to all the preschools in China. In order to determine whether different organizational structures or management models for preschools can moderate the relationship between leadership styles and teacher leadership, future research could broaden the scope of the participant to include various types of preschools in various regions of China.

Finally, in terms of research content, the mechanism of principal leadership style's influence on preschool teacher leadership has not been studied deeply enough. This research only explores the mediating variable of psychological capital, and many other variables that may affect the principal leadership style and preschool teacher leadership are not mentioned. There may even be chain mediating or moderating variables between the principal leadership style and preschool teacher leadership, which need to be further analyzed in future studies.

## Conclusion

This research, based on leadership-membership exchange theory, took preschool teachers in Guangdong Province of China as participants and analyzed the relationship and the underlying mechanisms of action between preschool principal leadership styles, preschool teacher leadership and psychological capital by distributing a large-scale questionnaire. First of all, the results showed that preschool teacher leadership and psychological capital were at moderate to high levels, with significant differences in terms of seniority and position. The principal leadership styles of preschool are mainly transformational leadership style and transactional leadership style. Secondly, transformational leadership style and transactional leadership style showed significant positive correlations with preschool teacher leadership, and laissez-faire leadership style showed no correlation with preschool teacher leadership. Preschool teacher leadership was significantly and positively correlated with psychological capital. Transformational leadership style and transactional leadership style were significantly and positively correlated with psychological capital, while laissez-faire leadership style was significantly and negatively correlated with psychological capital. Finally, Transformational leadership style can positively influence preschool teacher leadership directly and indirectly through psychological capital; transactional leadership style can only positively influence preschool teacher leadership indirectly through the mediating role of psychological capital. Laissez-faire can neither directly influence preschool teacher leadership nor indirectly influence preschool teacher leadership through the mediating role of psychological capital.

## Data availability statement

The raw data supporting the conclusions of this article will be made available by the authors, without undue reservation.

## Ethics statement

All procedures followed were in accordance with the ethical standards of the responsible Committee on Human Experimentation [Guangzhou University, Guangdong Province, China] and with the Helsinki Declaration of 1975, as revised in 2000. Written informed consent to participate in this study was provided by the participants.

## Author contributions

LZ designed the research and drafted the manuscript. TW and CL collected and extracted data for analysis. LL and PR provided important ideas and substantial feedback for the study and edited the manuscript. All authors have approved the final version of this article.

## Funding

This work was supported by the Project of Humanities and Social Sciences of Ministry of Education of the People's Republic of China [21YJC880092].

## Conflict of interest

The authors declare that the research was conducted in the absence of any commercial or financial relationships that could be construed as a potential conflict of interest.

## Publisher's note

All claims expressed in this article are solely those of the authors and do not necessarily represent those of their affiliated organizations, or those of the publisher, the editors and the reviewers. Any product that may be evaluated in this article, or claim that may be made by its manufacturer, is not guaranteed or endorsed by the publisher.
